# Enhanced recovery after surgery program focusing on chest tube management improves surgical recovery after video-assisted thoracoscopic surgery

**DOI:** 10.1186/s13019-024-02762-3

**Published:** 2024-04-20

**Authors:** Dan Yang, Xi Zheng

**Affiliations:** 1 Lung Cancer Center, West China Hospital, Sichuan University/West China School of Nursing, Chengdu, 610000 Sichuan China; 2 Department of Thoracic Surgery, West China Hospital, Sichuan University, No. 37 Guoxue Alley, Wuhou District, Chengdu, 610000 Sichuan China

**Keywords:** Video-assisted thoracoscopic surgery, Enhanced recovery after surgery, Lung cancer, Chest tube management, Postoperative complication

## Abstract

**Objective:**

Chest drainage is a standard procedure in thoracoscopic surgery for lung cancer. However, chest tube placement may deteriorate the ventilation capacity and increase difficulty of postoperative management of patients. The study investigated on the effects of enhanced recovery after surgery (ERAS) program focusing on chest tube management on surgical recovery of lung cancer patients.

**Methods:**

The study population consisted of 60 patients undergoing video-assisted thoracoscopic surgery (VATS) after implementation of ERAS program and another group of 60 patients undergoing VATS before implementation of ERAS program.

**Results:**

The mean time of first food intake was 12.9 h required for the ERAS group, which was significantly shorter than 18.4 h required for the control group (*p* < 0.0001). The mean time of out-of-bed activity was 14.2 h taken for the ERAS group, which was notably shorter than 22.8 h taken for the control group (*p* < 0.0001). The duration of chest tube placement was 68.6 h in the ERAS group, which was remarkably shorter than 92.8 h in the control group (*p* < 0.0001). The rate overall postoperative complications were notably lower in the ERAS group than in the control group (*p* = 0.018). The visual analogue score (VAS) scores on the second postoperative day exhibited significant differences between the ERAS group and the control group (*p* = 0.017). The patients in the ERAS group had a shorter hospitalization stay than those in the control group (*p* < 0.0001).

**Conclusion:**

The study suggests the ERAS program focusing on chest tube management could improve surgical recovery, remove patient chest tube earlier, and relieve patient pain after VATS.

## Introduction

Lung cancer still remains the leading cause of cancer-related death on a global scale while its incidence and mortality show geographical differences substantially due to varying patterns of well-recognized risk factors, such as tobacco smoking and air pollutants [1, 2]. According to the Global Cancer Statistics across 185 countries updated in 2020, lung cancer was expected to cause 11.4% of all new cancer diagnoses and 18.0% of all cancer-related deaths [3]. According to Cancer Statistics updated by the American Cancer Society in 2023, there will be approximately 238,340 newly diagnosed cases of lung cancer and 127,070 new deaths in the United State (US) [4]. Although only age and smoking history are recommended as current lung cancer screening guidelines by the US Preventive Services Task Force (USPSTF), individualized risk evaluation including environmental exposures, chronic lung disease, and family history should be constituted into lung cancer screening guidelines as recommended by the National Comprehensive Cancer Network and other society guidelines [5, 6]. For the vast majority of patients with early-stage lung cancer, surgical resection is still the standard of care and has revolutionized from a traditional open approach to a minimally invasive surgery technique mainly represented by video-assisted thoracoscopic surgery (VATS) [7]. VATS permits proven safety and feasibility, less surgical trauma, and shorter postoperative hospital days, which has become a mainstay of modern-day thoracic oncology practice for resection of early-stage lung cancers [8]. Nevertheless, this type of minimally invasive surgery still induces surgical stress and considerable postoperative complications, making postoperative management being a clinical challenge for lung cancer patients [9].

Enhanced recovery after surgery (ERAS) refers to a multimodal, multidisciplinary approach integrating various procedures patient’s initial referral through to discharge with a goal to minimize surgical stress, improve surgical outcomes, reduce postoperative complications, shorten hospital stay, and improve surgical productivity [10]. Over the last few years, a large volume of evolving clinical evidence has successfully confirmed improved surgical outcomes with safety in major surgical specialties including VATS [11–13]. Key elements to successfully implement ERAS protocols are the setting up of a dedicated team consisting of surgeons and surgical groups, preoperative counseling and nutrition, promotion of early oral intake, and early mobilization [14]. With regard to thoracic surgery to implement ERAS protocols, in addition to the utilization of minimally invasive approaches and standard postoperative care, proper chest tube management should be added due to the fact that chest tubes can induce pain, deteriorate the ventilation capacity, and delay mobilization [15], . Prolonged drainage and air leaks resulting from chest tube placement may lead to a substantial burden on hospitalization stay and outpatient resources, creating a critical need for a dedicated chest tube management, such as the setting up of chest tube monitoring clinic and development of a novel chest tube placement to improve postoperative care for patients after thoracic surgery [16, 17]. Early removal of the chest tube after VATS has been pursued to shorten length of hospital stays and most importantly, reduce postoperative morbidity without increasing the risk of complications [18]. Although the ERAS protocols have been implemented in VATS over the few years, optimization of chest tube management to achieve early removal of the chest tube remain to be explored [19]. The objectives of this study were to implement a standardized ERAS program for patients undergoing VATS, analyze the safety and feasibility of this program with a specific focus on chest tube management by assessing associated surgical recovery.

## Methods

### Study population

The study population consisted of 60 patients undergoing video-assisted thoracoscopic surgery (VATS) after implementation of ERAS program (performed 2022 and December 2022) and another group of 60 patients undergoing VATS before implementation of ERAS program (performed 2021 and December 2021). Inclusion criteria were: (i) the VATS was performed under general anesthesia with double lumen intubation; (ii) clinical stage I to IIIB NSCLC; (iii) single peripheral mass less than 4 cm, without tumor invasion of the chest wall or great vessels; (iv) the VATS was performed by the same pool of surgeons in the year; iv) no distant metastasis, confirmed by preoperative examination; and (v) aged ≥ 18 years. Exclusion criteria were (i) N2 or N3 clinical nodal involvement of disease; (ii) induction chemotherapy or neoadjuvant therapies before surgery; (iii) conversion to thoracotomy; (iv) previous history of thoracic surgery (any type) or chest trauma; or (v) severe chronic obstructive pulmonary disease, bronchial asthma, or interstitial lung disease.

### Surgical methods

Patients of two groups had an only difference that their postoperative management followed the ERAS program. If the patients had diabetes, their glucose levels were maintained at a mild elevation. If the patients had hypotension, their blood pressure were maintained 160/100 mmHg or below. All patients received double-lumen endotracheal intubation with single-lung ventilation in the lateral position on the healthy side and underwent uniportal VATS. An incision with a length of 3–5 cm was made, and the camera was placed on the upper edge of the incision. The intrathoracic tissues were dissociated and the lymph nodes were dissected. One chest tube was placed through the camera port after lobectomy. The chest tube was adjusted with side holes made between the diaphragm and the lung lobe to drain pleural effusion. After surgery, all patients were given multimodal analgesia.

### ERAS program

We developed a VATS-specific ERAS program as previously described [14, 20, 21] and modified this program with a specific focus on chest tube management. All related medical staff members (thoracic surgeons, anesthesiologists, and nutritionists) and a specifically ERAS designated study nurse received specific instructions regarding the modified ERAS program. Comments and advices from all these staff and nurses were collected and integrated into the final program prior to study commencement. Table 1 itemizes the key elements of the VATS-specific ERAS program and Table 2 lists the key elements of chest tube management.

**Table 1:** Scheme of key elements of the VATS-specific ERAS program

	Control	ERAS
Preoperative		
Consultation	No standardized information	An exhaustive preoperative patient information; psychological care
Education	Video	Standardized education protocols; information booklet with daily goals; family member engagement; smoking cessation; nutritional advice; incentive spirometer every 1 h
Intraoperative		
Pain control	Epidural catheter or intercostal; halogenated; anesthetics gases/propofol	Intercostal blocks
Chest tube diameter	28 F	24 F
Fluid control	Intravenously administration of rehydration 1800 ml	Avoidance of sodium/fluid overload; goal-directed fluid therapy
Postoperative		
Analgesia	Patient controlled analgesia (dizosin)	Patient controlled analgesia (celecoxib)
Activity	Routine	Early mobilization; out of bed 2 h on the day of surgery and 6 h per day until discharge; respiratory physiotherapy and incentive spirometer every 1 h
Nourishment	Oral intake on the 1st day after surgery or anal exhaust	Promotion of early oral intake (water and soft foods) after surgery
Urinary catheter	Yes	No
Chest tube management	Water seal (passive suction); the tubes removed after less than 250 mL/24 h of drainage and no air flow	The tube removed after less than 400 mL/24 h of drainage and no air flow; detailed in Table 2
Discharge	Meeting discharged criteria	Meeting discharged criteria; a telephone follow-up with health education at 1 month, 3 months, and 6 months after surgery

**Table 2:** Perioperative and postoperative orders for chest tube management contained in this ERAS program

Key element	Detail
Psychological care	Education for chest drainage before surgery
Position for bed rest	Semi-recumbent position switch from 30° to 45°
Drainage observation	Nature, quantity, color, and speed of drainage
Skin care surrounding the tube	Prevention of redness, swelling, blisters, allergy, and damage in the skin
Tube fixation	Prevention of detachment, twisting, folding, and compression
Removal of drain	< 400 mL/24 h and no air flow; good pulmonary dilation by chest x-ray; clear breathing sound during auscultation

### Data collection and variable definitions

Preoperative patient characteristics were recorded: age, sex, body mass index (BMI), education level, American Society of Anesthesiologists (ASA) grade, comorbidities [hypertension, diabetes, and chronic obstructive pulmonary disease (COPD)], arterial oxygen partial pressure (PaO_2_), arterial carbon dioxide partial pressure (PaCO_2_), forced expiratory volume in 1 s (FEV1), diffusing capacity of the lung for carbon dioxide (DLCO), pathologic stage, and lesion locations.

Postoperative variables were recorded: surgical duration, the time of first food intake, the time of out-of-bed activity, duration of chest tube placement, pain scores, postoperative complications, readmission, and hospital length of stay (LOS).

The patient pain was evaluated by the visual analogue score (VAS) that is an assessment tool used to quantify pain ranging from 1 to 10, with a higher score indicating a greater severity.

Postoperative complications were categorized according to the ClavienDindo classification which is adapted for thoracic surgery while considering grade I (wound infection opened at bedside and pneumonia) and grade II (pneumonia) as minor, grade III (atelectasis requiring a bronchoscopy) and grade IV (respiratory failure requiring intubation) as major complications, and grade V as 30-day postoperative mortality. Postoperative pneumonia is defined as abnormal radiographic findings including new infiltrations evidenced on postoperative chest x-ray or computed tomography and the following one or more symptoms postoperatively during hospitalization: (i) new or progressive respiratory symptoms, such as coughing and expectoration; (ii) a vital sign of fever > 38 °C or hypothermia; (iii) evidence of lung consolidation signs or moist rale based on physical examinations; (iv) white cell counts or < 4 × 10^9^/L or > 10 × 10^9^ /L, or a new elevation in the level of C-reactive protein; and (v) positive results of blood culture or sputum [22]. The degree of juxtapleural consolidation was scored as 0 indicating no consolidation, 1 indicating minimal juxtapleural consolidation, 2 indicating small-sized consolidation, and 3 indicating large-sized consolidation. Significant atelectasis was defined by a consolidation score of at least 2 in any region [23].

### Statistical analysis

The outcomes were reported as mean ± standard deviation (s.d.) for measurement variables normally distributed, as medians with interquartile ranges for measurement variables not normally distributed, and as counts (%) for categorical variables. Student’s t-test, Mann-Whitney test, and chi-square test was used for statistical analysis, with *p* < 0.05 as the level of significance for all tests in the GraphPad prism 8.0 (GraphPad Software, San Diego, CA, USA).

## Results

### Demographic and clinical characteristics of patients

Demographic and clinical characteristics of patients in the ERAS group and control group were presented in Table 3. Two groups were comparable for their outcome analysis as no significant differences noted in age, sex, BMI, education level, ASA grade, comorbidities, PaO_2_, PaCO_2_, FEV1, DLCO, pathologic stage, and lesion locations.

**Table 3:** Demographic and clinical characteristics of patients

Characteristics	ERAS (*n* = 60)	Control (*n* = 60)	p
Age (year, mean ± s.d.)	54.1 ± 10.6	52.4 ± 10.3	0.357
Sex (male/female)	24/36	28/32	0.461
BMI (kg/m^2^)	22.5 ± 3.3	22.8 ± 3.1	0.609
Education level (year)	10.6 ± 2.8	10.2 ± 2.6	0.419
ASA (II/III)	31/29	34/26	0.514
Comorbidities			
Hypertension (yes/no)	33/27	26/34	0.201
Diabetes (yes/no)	10/50	12/48	0.637
COPD (yes/no)	18/42	21/39	0.559
PaO_2_ (mm Hg, mean ± s.d.)	87.9 ± 6.8	87.4 ± 6.4	0.679
PaCO_2_ (mm Hg, mean ± s.d.)	40.4 ± 2.5	40.2 ± 2.3	0.649
FEV1 (%, mean ± s.d.)	92.4 ± 16.1	93.1 ± 15.8	0.811
DLCO (%, mean ± s.d.)	81.2 ± 22.7	80.6 ± 23.0	0.886
Pathologic stage			0.132
IIA	22	28	
IIB	11	16	
IIIA	25	16	
IIIB	2	0	
Resection (lobectomy/segmentectomy)	35/25	37/23	0.709

BMI, body mass index; COPD, chronic obstructive pulmonary disease; PaO_2_, arterial oxygen partial pressure; PaCO_2_, arterial carbon dioxide partial pressure; FEV1, forced expiratory volume in 1 s; DLCO, diffusing capacity of the lung for carbon dioxide; the value of p yielded by unpaired t test and chi-square test, respectively.

### Effects of the ERAS program on postoperative management of patients

**Fig. 1:**
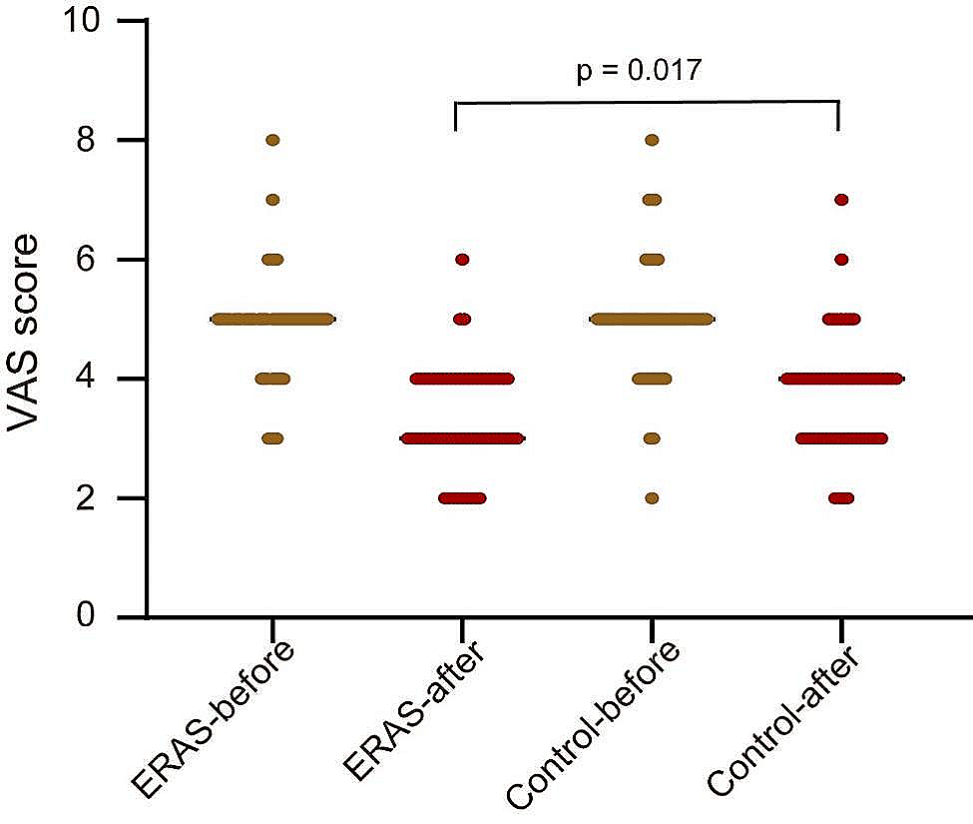
The VAS scores of patients on the first preoperative day and the second postoperative day between the ERAS group and the control group. Mann-Whitney test was used for statistical analysis

The patients in the ERAS group were compared with those in the control group with regard to surgical duration, lymph node yield, the time of first food intake, the time of out-of-bed activity, and duration of chest tube placement (Table 4). The surgical duration did not differ between two groups (*p* > 0.05). The mean time of first food intake was 12.9 h required for the ERAS group, which was significantly shorter than 18.4 h required for the control group (*p* < 0.0001). The mean time of out-of-bed activity was 14.2 h taken for the ERAS group, which was notably shorter than 22.8 h taken for the control group (*p* < 0.0001). The duration of chest tube placement was 68.6 h in the ERAS group, which was remarkably shorter than 92.8 h in the control group (*p* < 0.0001). Two groups did not differ in VAS scores on the first preoperative day. The VAS scores on the second postoperative day exhibited significant differences between the ERAS group and the control group (*p* = 0.017, Fig. 1). These data suggest that the implementation of the ERAS program could improve surgical recovery, remove patient chest tube earlier, and relieved patient pain after VATS.

**Table 4:** Effects of the ERAS program on postoperative management of patients

Variable	ERAS (*n* = 60)	Control (*n* = 60)	p
Surgical duration (min, mean ± s.d.)	159.3 ± 30.2	168.5 ± 30.8	0.101
Time of first food intake (h, mean ± s.d.)	12.9 ± 7.1	18.4 ± 7.5	< 0.0001
Time of out-of-bed activity (h, mean ± s.d.)	14.2 ± 8.0	22.8 ± 13.3	< 0.0001
Duration of chest tube placement (h, mean ± s.d.)	68.6 ± 36.7	92.8 ± 50.3	< 0.0001

### Effects of the ERAS program on postoperative complications of patients

No case of 30-day mortality occurred in the ERAS group and control group. The ERAS group had no patient requiring a readmission. The control group had 1 patient (1.6%) requiring a readmission to redo VATS. There were 2 cases of pneumonia, 1 cases of air leak > 7 days, 1 case of atrial fibrillation, 1 cases of urinary retention, and 1 cases of pneumothorax in the ERAS group, with a postoperative morbidity of 10.0%. There were 5 cases of pneumonia, 3 cases of air leak > 7 days, 2 cases of atelectasis, 2 case of atrial fibrillation, 2 cases of urinary retention, 1 case of chylothorax, and 1 cases of pneumothorax in the control group, with a postoperative morbidity of 26.7%. The rates overall postoperative morbidity and minor complications were notably lower in the ERAS group than in the control group (Table 5, *p* = 0.018 and *p* = 0.024). These data suggest that the implementation of the ERAS program could prevent the incidence of postoperative complications for patients undergoing VATS.

**Table 5:** Effects of the ERAS program on postoperative complications of patients

Variable	ERAS (*n* = 60)	Control (*n* = 60)	p
Readmission	0	1	0.559
Redo VATS	0	1	0.315
Minor complication	5 (8.33%)	14 (23.33%)	0.024
pneumonia	2	5	0.436
Air leak > 7 days	1	3	0.611
Atelectasis	0	2	0.476
Atrial fibrillation	1	2	1.000
Urinary retention	1	2	1.000
Major complication	1 (1.6%)	2 (3.3%)	0.559
Chylothorax	0	1	1.000
Pneumothorax	1	1	1.000
Postoperative morbidity	6 (10.0%)	16 (26.7%)	0.018

### Effects of the ERAS program on LOS of patients undergoing VATS

The median LOS of patients undergoing VATS was 3 d (range: 2–5 d) in the ERAS group and 5 days (range: 4–6 d) in the control group. The patients in the ERAS group had a shorter LOS than those in the control group (*p* < 0.0001). These data suggest that the implementation of the ERAS program could shorten the LOS of patients undergoing VATS.

## Discussion

Although VATS is a minimally invasive procedure that permits relatively fast recovery, the use of chest tubes for drainage of the thorax postoperatively causes moderate to severe postoperative pain, increased risk of infection, and prolonged length of hospital stay [24]. The current issues and challenges of chest tube placement during VATS refer to (i) a suitable size and type of chest tube to prevent blockage; (ii) the appropriate number of chest tubes to achieve effective drainage effectiveness and reduced postoperative pain; (iii) utilization of a digital classification system routinely to reduce the duration of air leakage; (iv) optimized the drainage volume during the period for early chest tube removal; and (v) even without drainage-tube placement in some strictly selected patients [25–28]. However, chest tube placement still remains a standard procedure during VATS in multiple medical centers, and evidence-based chest tube management need to be developed and widely adopted. This study suggests the implementation of a VATS-specific ERAS program that was modified with a specific focus on chest tube placement could improve surgical recovery and remove patient chest tube earlier to achieve reduced hospital stay compared to usual care and it did not increase readmission rates.

Preoperative consultation with a clear comprehensible explanation of what is to happen across the entire hospitalization process was one of the ERAS principles to alleviate depression, anxiety, and surgical fear of patients [29]. A rational yet nonaggressive goal-directed fluid therapy as an intraoperative element of the ERAS protocols could reduce the incidence of postoperative complications in the intestines and prevent venous thromboembolism [30, 31]. Promotion of oral intake and early mobilization as two core elements of postoperative part of ERAS protocols, aiming at faster recovery of bowel function, lower rates of infectious complications, and shorter hospital stay [32]. Our results showed that the time of first food intake and the time of out-of-bed activity were significantly shorter in the patients undergoing VATS after implementation of ERAS program compared to those before implementation of ERAS program, indicating a better surgical recovery without adding the risk of complications offered by the ERAS program in VATS.

Traditionally, placement of two chest tubes (one in the apical position and a second in the basal position) was conducted to enhance expansion of the residual lungs, whereas increasing numbers of studies reported one chest tube placement is applicable in normal clinical situations as adequate drainage was obtained with less pain and shorter hospital stay [18]. Recently, use of thinner chest tube is investigated to achieve efficient fluid drainage and air evacuation [33]. However, Clark et al. [34] reported this thin and flexible tube became completely occluded resulting in life-threatening hypovolemic shock after thoracic surgery. This indicates that the use of thinner chest tube is applicable in normal clinical situations, but adding risk of postoperative bleeding and infection should be taken into account. Icard et al. [35] examined the feasibility and safety of a single 24 F Blake drainage in 100 consecutive patients undergoing lobectomy or wedge resection and suggested it can be considered as an acceptable option due to the fact that the flexible quality of the drain improved comfort of the operated patients. Chestovich et al. [36] tested flowrates of chest tubes among 20 F, 24 F, 28 F, 32 F, and 36 F, and found smaller tubes being slower and more variable and larger tubes showing only slightly higher flowrates. A single 28 F chest tube exhibited high and consistent velocity, keeping a good balance of reasonable size and high flowrate. Chest tube management was the postoperative core of the ERAS program in our study, which was evolved with education for chest drainage, position for bed rest, drainage observation (nature, quantity, color, and speed of drainage), skin care surrounding the tube, tube fixation, appropriate criteria of chest tube removal. Our results showed the patients undergoing VATS after implementation of ERAS program presented shorter chest tube days and hospital LOS than those before implementation of ERAS program, indicating the ERAS program modified with chest tube management could reduce chest tube days and hospital LOS without adding risk of infection.

Several limitations should be noted when the present data were interpretated. Retrospective data collection for outcome analysis might result in incomplete identification of postoperative events. Further investigations of the ERAS program focusing on chest tube management should be implemented in randomized clinical trials. Additional limitation was relatively sample size in single center. Considering relatively small sample size, the effects of smaller size tube on surgical recovery and the incidence of pneumonia may be further analyzed in a large-scale study. Although the patients undergoing VATS are likely to obtain cost benefits from this ERAS program, we fail to undertake any health costing analyses due to under-estimate of hospitalization costs of the control group. It is possible that the particular care by nursing staff, such as use of incentive spirometer, early mobilization and respiratory physiotherapy would increase costs. However, previous evidence showed that the ERAS program for VATS anatomical lung resection is cost-effective and is associated with a lower complication rate and a shorter postoperative hospitalization [21]. Although the particular care in the ERAS group would increase costs, management of complications and longer hospitalization would also increase costs for the control group. All in all, further prospective studies are required to clearly estimate the costs of the ERAS program compared to the transitional nursing strategies.

In conclusion, our results indicate that the implementation of VATS-specific ERAS program with a specific focus on chest tube management was associated with improved surgical recovery, reduced pain, lesser postoperative complications, and a shorter hospitalization stay.

## Data Availability

The data used for the study are available in the present study.

## References

[1] Leiter A, Veluswamy RR, Wisnivesky JP (2023). The global burden of lung cancer: current status and future trends. Nat Rev Clin Oncol.

[2] Hill W, Lim EL, Weeden CE, Lee C, Augustine M, Chen K, Kuan FC, Marongiu F, Evans EJ, Moore DA (2023). Lung adenocarcinoma promotion by air pollutants. Nature.

[3] Sung H, Ferlay J, Siegel RL, Laversanne M, Soerjomataram I, Jemal A, Bray F (2021). Global Cancer statistics 2020: GLOBOCAN estimates of incidence and Mortality Worldwide for 36 cancers in 185 countries. CA Cancer J Clin.

[4] Siegel RL, Miller KD, Wagle NS, Jemal A (2023). Cancer statistics, 2023. CA Cancer J Clin.

[5] Wu JT, Wakelee HA, Han SS (2023). Optimizing Lung Cancer Screening with Risk Prediction: current challenges and the emerging role of biomarkers. J Clin Oncol.

[6] Adams SJ, Stone E, Baldwin DR, Vliegenthart R, Lee P, Fintelmann FJ (2023). Lung cancer screening. Lancet.

[7] Ujiie H, Gregor A, Yasufuku K (2019). Minimally invasive surgical approaches for lung cancer. Expert Rev Respir Med.

[8] Sihoe ADL (2020). Video-assisted thoracoscopic surgery as the gold standard for lung cancer surgery. Respirology.

[9] Jiao W, Zhao Y, Wang M, Wang Z, Yang R, Wang Y, Luo Y, Shen Y (2015). A retrospective study of diaphragmatic motion, pulmonary function, and quality-of-life following video-assisted thoracoscopic lobectomy in patients with nonsmall cell lung cancer. Indian J Cancer.

[10] Draeger TB, Gibson VR, Fernandes G, Andaz SK (2021). Enhanced recovery after thoracic surgery (ERATS). Heart Lung Circ.

[11] Ripolles-Melchor J, Abad-Motos A, Zorrilla-Vaca A (2022). Enhanced recovery after surgery (ERAS) in Surgical Oncology. Curr Oncol Rep.

[12] Huang L, Kehlet H, Petersen RH (2022). Functional recovery after discharge in enhanced recovery video-assisted thoracoscopic lobectomy: a pilot prospective cohort study. Anaesthesia.

[13] Huang L, Frandsen MN, Kehlet H, Petersen RH. Days alive and out of hospital after enhanced recovery video-assisted thoracoscopic surgery lobectomy. Eur J Cardiothorac Surg 2022, 62(3). 10.1093/ejcts/ezac148 35234866

[14] Gonfiotti A, Viggiano D, Bongiolatti S, Bertolaccini L, Solli P, Bertani A, Voltolini L, Crisci R, Droghetti A (2018). Enhanced recovery after surgery (ERAS((R))) in thoracic surgical oncology. Future Oncol.

[15] Anderson D, Chen SA, Godoy LA, Brown LM, Cooke DT (2022). Comprehensive Review of Chest Tube Management: a review. JAMA Surg.

[16] Tcherveniakov P, De Siqueira J, Milton R, Papagiannopoulos K (2012). Ward-based, nurse-led, outpatient chest tube management: analysis of impact, cost-effectiveness and patient safety. Eur J Cardiothorac Surg.

[17] Xu Y, Luo J, Ge QY, Cong ZZ, Jiang ZS, Diao YF, Huang HR, Wei W, Shen Y (2023). Safety and feasibility of a novel chest tube placement in uniportal video-assisted thoracoscopic surgery for non-small cell lung cancer. Thorac Cancer.

[18] Gottgens KW, Siebenga J, Belgers EH, van Huijstee PJ, Bollen EC (2011). Early removal of the chest tube after complete video-assisted thoracoscopic lobectomies. Eur J Cardiothorac Surg.

[19] Deng B, Qian K, Zhou JH, Tan QY, Wang RW (2017). Optimization of Chest Tube Management to Expedite Rehabilitation of Lung Cancer patients after Video-assisted thoracic surgery: a Meta-analysis and systematic review. World J Surg.

[20] Huang H, Ma H, Chen S (2018). Enhanced recovery after surgery using uniportal video-assisted thoracic surgery for lung cancer: a preliminary study. Thorac Cancer.

[21] Gonzalez M, Abdelnour-Berchtold E, Perentes JY, Doucet V, Zellweger M, Marcucci C, Ris HB, Krueger T, Gronchi F (2018). An enhanced recovery after surgery program for video-assisted thoracoscopic surgery anatomical lung resections is cost-effective. J Thorac Dis.

[22] Cakir Edis E, Hatipoglu ON, Yilmam I, Eker A, Tansel O, Sut N (2009). Hospital-acquired pneumonia developed in non-intensive care units. Respiration.

[23] Lee JH, Choi S, Ji SH, Jang YE, Kim EH, Kim HS, Kim JT (2020). Effect of an ultrasound-guided lung recruitment manoeuvre on postoperative atelectasis in children: a randomised controlled trial. Eur J Anaesthesiol.

[24] Jones CW, Rodriguez RD, Griffin RL, McGwin G, Jansen JO, Kerby JD, Bosarge PL (2019). Complications Associated with Placement of chest tubes: a Trauma System Perspective. J Surg Res.

[25] Wei S, Zhang G, Ma J, Nong L, Zhang J, Zhong W, Cui J (2022). Randomized controlled trial of an alternative drainage strategy vs routine chest tube insertion for postoperative pain after thoracoscopic wedge resection. BMC Anesthesiol.

[26] Song Y, Zheng C, Zhou S, Cui H, Wang J, Wang J, Wang W, Liu L, Liu J (2021). The application analysis of 8F ultrafine chest drainage tube for thoracoscopic lobectomy of lung cancer. J Cardiothorac Surg.

[27] Nakanishi R, Fujino Y, Kato M, Miura T, Yasuda M, Oda R, Yokota K, Okuda K, Haneda H (2018). Early chest tube removal after thoracoscopic lobectomy with the aid of an additional thin tube: a prospective multi-institutional study. Gen Thorac Cardiovasc Surg.

[28] Ueda K, Hayashi M, Tanaka T, Hamano K (2013). Omitting chest tube drainage after thoracoscopic major lung resection. Eur J Cardiothorac Surg.

[29] Bian T, Shao H, Zhou Y, Huang Y, Song Y (2021). Does psychological distress influence postoperative satisfaction and outcomes in patients undergoing total knee arthroplasty? A prospective cohort study. BMC Musculoskelet Disord.

[30] Taylor JS, Iniesta MD, Zorrilla-Vaca A, Cain KE, Lasala JD, Mena GE, Meyer LA, Ramirez PT (2023). Rate of venous thromboembolism on an enhanced recovery program after gynecologic surgery. Am J Obstet Gynecol.

[31] Minto G, Scott MJ, Miller TE (2015). Monitoring needs and goal-directed fluid therapy within an enhanced recovery program. Anesthesiol Clin.

[32] Charoenkwan K, Matovinovic E (2014). Early versus delayed oral fluids and food for reducing complications after major abdominal gynaecologic surgery. Cochrane Database Syst Rev.

[33] Nakamura H, Taniguchi Y, Miwa K, Adachi Y, Fujioka S, Haruki T (2009). The 19Fr Blake drain versus the 28Fr conventional drain after a lobectomy for lung cancer. Thorac Cardiovasc Surg.

[34] Clark G, Licker M, Bertin D, Spiliopoulos A (2007). Small size new silastic drains: life-threatening hypovolemic shock after thoracic surgery associated with a non-functioning chest tube. Eur J Cardiothorac Surg.

[35] Icard P, Chautard J, Zhang X, Juanico M, Bichi S, Lerochais JP, Flais F (2006). A single 24F Blake drain after wedge resection or lobectomy: a study on 100 consecutive cases. Eur J Cardiothorac Surg.

[36] Chestovich PJ, Jennings CS, Fraser DR, Ingalls NK, Morrissey SL, Kuhls DA, Fildes JJ (2020). Too big, too small or just right? Why the 28 French chest tube is the best size. J Surg Res.

